# Author Correction: The dynamics of plant nutation

**DOI:** 10.1038/s41598-021-87666-1

**Published:** 2021-04-14

**Authors:** Vicente Raja, Paula L. Silva, Roghaieh Holghoomi, Paco Calvo

**Affiliations:** 1 Rotman Institute of Philosophy, Western University, London, Canada; 2 Department of Psychology, University of Cincinnati, Cincinnati, USA; 3 Department of Biology, Faculty of Science, Urmia University, Urmia, Iran; 4 Minimal Intelligence Lab, University of Murcia, Murcia, Spain

Correction to: *Scientific Reports* 10.1038/s41598-020-76588-z, published online 10 November 2020.

The original version of this Article contained a repeated error for pole height. The text,

“0.90 cm”.

now reads:

“0.90 m”.

This has been corrected in the PDF and HTML versions of the Article.

