# Supplementary material for: The dynamics of plant nutation
Source: Sci Rep. 2020 Nov 10;10:19465. doi: 10.1038/s41598-020-76588-z (PMC7655864; doi:10.1038/s41598-020-76588-z)
Supplement: Supplementary file 2 — Supplementary Information 2. [file 41598_2020_76588_MOESM2_ESM.pdf]

The Dynamics of Plant Nutation

Vicente Raja[1*], Paula L. Silva[2], Roghaieh Holghoomi[3, 4] and Paco Calvo[4]

[1] Rotman Institute of Philosophy, Western University, Canada – *E-mail: vgalian@uwo.ca
[2] Department of Psychology, University of Cincinnati, USA
[3] Department of Biology, Faculty of Science, University of Urmia, Iran
[4] Minimal Intelligence Lab, University of Murcia, Spain

- Supplementary Figure F1
- Supplementary Data D1 – Dynamical and Statistical Analyses (separate document ‘Supplementary Dataset D1 – Dynamical and Statistical Analyses.xlsx’)
- Supplementary Data D2 – Statistical Models
- Supplementary Video V1 – Pair 2 Pole Condition (‘Supplementary Video V1.mov’)**
- Supplementary Video V2 – Pair 2 No Pole Condition (‘Supplementary Video V2.mov’)**

**IMPORTANT: Better quality .AVI videos are available upon request.

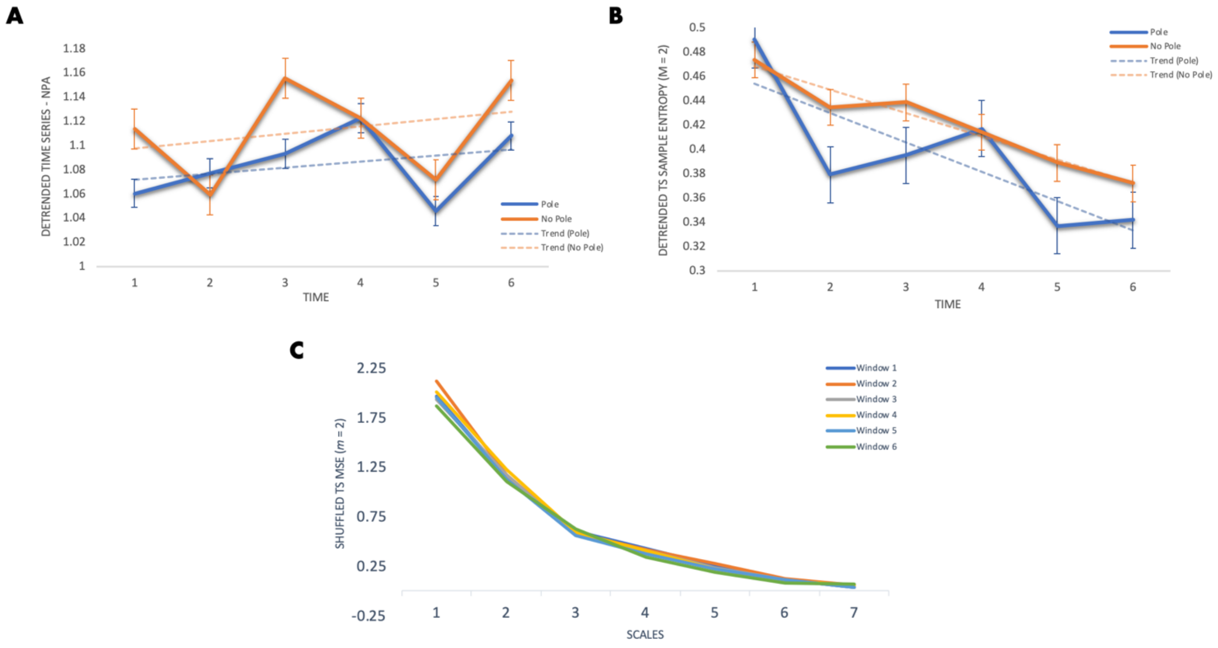

Supplementary Figure 1. (A) NPA Analysis of detrended time series. Average values of NPA per windows 1 to 6 including standard errors. Solid blue line plots average NPA values for plants in pole condition and dashed blue line plots its linear trend. Solid orange line plots mean NPA values for plants in no-pole condition and dashed orange line plots its linear trend. The average NPA values and their trends for both conditions are almost indistinguishable from the produced by the original data (see Figure 3A). **(B)** SampEn Analysis of detrended time series. Average SampEn values per windows 1 to 6 and standard errors for $m = 2$, $r = .25$. Solid blue line plots average SampEn values for plants in pole condition and dashed blue line plots its linear trend. Solid orange line plots average SampEn values for plants in no-pole condition and dashed orange line plots its linear trend. The average SampEn values and their trends for both conditions are almost indistinguishable from the produced by the original data (see Figure 3B). **(C)** EMD-based MSE ($m = 2$, $r = .25$) for the surrogate shuffled time series of the pole condition including all windows and scales. The effect observed in the EMD-based MSE ($m = 2$, $r = .25$) for the original time series of the pole condition disappears (see Figure 4B-left).

Supplementary Data D2 – Statistical Models

Software Package

To analyze the data software package R studio, version 3.6.3, was used.

Packages used in R Studio

Package 'lme4' (Linear Mixed Effects Models)

Full Statistical Results

Normalized Peak Acceleration (NPA)

- Deviance comparison (-2 Log Likelihood; -2LL hereafter) between the full model (includes 2-ways interactions; NPA.F) and the reduced model (no interactions; NPA.R):

```
Data: Data.NPA_SE
Models:
NPA.R: NPA ~ Pole + Window + (1 | Pair/Window)
NPA.F: NPA ~ Pole * Window + (1 | Pair/Window)
```

	npar	AIC	BIC	logLik	deviance	Chisq	Df	Pr(>Chisq)
NPA.R	7	-200.89	-181.68	107.45	-214.89			
NPA.F	8	-198.89	-176.93	107.45	-214.89	0	1	0.9967

- Deviance comparison (-2LL) between the reduced model (NPA.R) and the model that only includes time (NPA.W):

```
Data: Data.NPA_SE
Models:
NPA.W: NPA ~ Window + (1 | Pair/Window)
NPA.R: NPA ~ Pole + Window + (1 | Pair/Window)
```

	npar	AIC	BIC	logLik	deviance	Chisq	Df	Pr(>Chisq)
NPA.W	6	-201.43	-184.96	106.71	-213.43			
NPA.R	7	-200.89	-181.68	107.45	-214.89	1.4649	1	0.2262

- Deviance comparison (-2LL) between the reduced model (NPA.R) and the model that only includes pole (NPA.P):

```
Data: Data.NPA_SE
Models:
NPA.P: NPA ~ Pole + (1 | Pair/Window)
NPA.R: NPA ~ Pole + Window + (1 | Pair/Window)
```

	npar	AIC	BIC	logLik	deviance	Chisq	Df	Pr(>Chisq)
NPA.P	6	-201.49	-185.02	106.75	-213.49			
NPA.R	7	-200.89	-181.68	107.45	-214.89	1.402	1	0.2364

Single-Scale Sample Entropy (SampEn)

- Deviance comparison (-2LL) between the full model (includes 2-ways interactions; SampEn.F) and the reduced model (no interactions; SampEn.R):

```
Data: Data.NPA_SE
Models:
SampEn.R: SE ~ Pole + Window + (1 | Pair/Window)
SampEn.F: SE ~ Pole * Window + (1 | Pair/Window)

          npar   AIC      BIC      logLik   deviance   Chisq   Df  Pr(>Chisq)
SampEn.R    6   -223.78  -207.37  117.89   -235.78
SampEn.F    7   -222.74  -203.58  118.37   -236.74    0.9512  1   0.3294
```

- Deviance comparison (-2LL) between the reduced model (SampEn.R) and the model than only includes time (SampEn.W):

```
Data: Data.NPA_SE
Models:
SampEn.W: SE ~ Window + (1 | Pair/Window)
SampEn.R: SE ~ Pole + Window + (1 | Pair/Window)

          npar   AIC      BIC      logLik   deviance   Chisq   Df  Pr(>Chisq)
SampEn.W    5   -220.88  -207.20  115.44   -230.88
SampEn.R    6   -223.78  -207.37  117.89   -235.78    4.9049  1   0.02678 *
```

- Deviance comparison (-2LL) between the reduced model (SampEn.R) and the model than only includes pole (SampEn.P):

```
Data: Data.NPA_SE
Models:
SampEn.P: SE ~ Pole + (1 | Pair/Window)
SampEn.R: SE ~ Pole + Window + (1 | Pair/Window)

          npar   AIC      BIC      logLik   deviance   Chisq   Df  Pr(>Chisq)
SampEn.P    5   -210.81  -197.13  110.41   -220.81
SampEn.R    6   -223.78  -207.37  117.89   -235.78    14.973  1   0.0001091 ***
```

Multiscale Sample Entropy (MSE)

- Deviance comparison (-2LL) between the full model (includes 3-ways interactions; MSE.F) and the reduced model (includes 2-ways interactions; MSE.R):

```
Data: Data.MSE
Models:
MSE.R: SE ~ Pole * Window + Pole * Scale + Window * Scale + (1 | Pair/Window) +
          (1 | Plant/Scale)
MSE.F: SE ~ Pole * Window * Scale + (1 | Pair/Window) + (1 | Plant/Scale)

        npar   AIC      BIC      logLik   deviance   Chisq    Df   Pr(>Chisq)
MSE.R    12   -1547.3  -1492.7   785.67   -1571.3
MSE.F    13   -1551.0  -1491.8   788.50   -1577.0    5.6645   1    0.01731 *
```

- Deviance comparison (-2LL) between the full model for scales 2 and 2 + windows 2, 3 and 6 (includes 2-ways interactions; MSE.F.Focus) and the reduced model (no interactions; MSE.R.Focus):

```
Data: Focus.MSE
Models:
MSE.R.Focus: SE ~ Pole + Scale + Window + (1 | Pair/Window)
MSE.F.Focus: SE ~ Pole * Scale + Window + (1 | Pair/Window)

              npar   AIC       BIC      logLik   deviance  Chisq    Df   Pr(>Chisq)
MSE.R.Focus     7   -187.75   -168.24   100.88   -201.75
MSE.F.Focus     8   -190.11   -167.81   103.06   -206.11   4.3589   1    0.03682 *
```

- Follow-up analysis of variance (ANOVA) of average SampEn values between scales 2 and 3 for the no pole condition (Anova.NP):

```
summary(Anova.NP)
            Df   Sum Sq     Mean Sq    F value   Pr(>F)
Scale        1   0.018598   0.018598   60.69     0.00146 **
Residuals    4   0.001226   0.000306
```

- Follow-up analysis of variance (ANOVA) of average SampEn values between scales 2 and 3 for the pole condition (Anova.P):

```
summary(Anova.P)
            Df   Sum Sq     Mean Sq    F value   Pr(>F)
Scale        1   0.00199    0.001990   1.28      0.321
Residuals    4   0.00622    0.001555
```
